# Survival time to complications of congestive heart failure patients at Felege Hiwot comprehensive specialized referral hospital, Bahir Dar, Ethiopia

**DOI:** 10.1371/journal.pone.0276440

**Published:** 2022-10-20

**Authors:** Nuru Mohammed Hussen, Demeke Lakew Workie, Hailegebrael Birhan Biresaw

**Affiliations:** 1 Department of Statistics, Samara University, Samara, Ethiopia; 2 Department of Statistics, Bahir Dar University, Bahir Dar, Ethiopia; 3 Department of Statistics, Debre Tabor University, Debre Tabor, Ethiopia; University of Siena: Universita degli Studi di Siena, ITALY

## Abstract

**Backgrounds:**

Congestive heart failure is a serious chronic condition when the heart’s muscles become too damaged and a condition in which one or both ventricles cannot pump sufficient blood to meet the metabolic needs of the body. This study aimed to identify factors affecting the complications time of congestive heart failure patients treated from January 2016 to December 2019 at Felege Hiwot comprehensive specialized referral hospital in Bahir Dar, Ethiopia.

**Methods:**

A hospital-based retrospective data collection was collected from the medical charts of 218 randomly selected congestive heart failure patients. The Kaplan-Meier curve and the Cox proportional hazards model were used to compare and identify the factors associated with time to complication in patients with congestive heart failure.

**Results:**

The median complication time of congestive heart failure patients was 22 months [95% CI: 21.98–28.01]. About 194 (88.99%) of the patients were complicated. The Kaplan-Meier curve depicts the survival probability of complicated patients decreasing as the complication time increases. The hazard ratios for serum sodium concentration, left ventricular ejection fraction, patients from rural areas, age of patients, serum hemoglobin concentration, and New York heart association classes I, II, and III were given 0.94 [95% CI: 0.90–1.00], 0.74 [95% CI: 0.65–0.85], 0.75 [95% CI: 0.68–0.84], 1.28 [95% CI: 1.12–1.46], 0.89 [95% CI: 0.85–0.94], 0.44 [95% CI: 0.36–0.53], 0.54 [95% CI: 0.47–0.62] and 0.73 [95% CI: 0.65–0.81] respectively, and they are statistically associated with the complication time of congestive heart failure patients.

**Conclusions:**

The median complication time of congestive heart failure patients was 22 months. This study strongly suggests that healthcare awareness should be strengthened earlier about the potential complications for patients with lower serum sodium concentrations below the threshold and aged congestive heart failure patients to reduce the risk of developing complications.

## Introduction

Congestive heart failure (CHF) is a chronic condition that happens when the heart’s muscles become too damaged and is a condition in which one or both ventricles cannot pump sufficient blood to meet the metabolic needs of the body. In the presence of heart failure, the heart still works, but the basic organs do not get enough blood and oxygen because the supply is insufficient [1]. Heart failure has been a chronic disease and a major public health problem for centuries due to its significant contribution to the global health burden. Its burden is on around 26 million people worldwide [2]. It is a serious worldwide health problem with high re-hospitalization and mortality rates [3]. The prevalence of heart failure has shown a dramatic increase over the past decades, and it is expected that there will be further increments due to the higher life expectancy of western societies [4]. Projections are even more alarming since the total cost of HF is expected to increase by 127% between 2012 and 2030 [5].

Every year in the USA, there are still 915,000 new cases of CHF, accounting for an incidence approaching 10 per 1,000 populations after 65 years of age. It is the primary or secondary diagnosis for over 2.4 million patients who are hospitalized and is responsible for nearly 300,000 annual deaths. The frequency of incidence of CHF is similar among men and women, and African-Americans are 1.5 times more likely to develop heart failure than people of any race with pale skin [6].

In Africa, CHF was more common in younger age groups, with most cases recorded around the fifth and sixth decades, and it is not a disease of the elderly in Sub-Saharan Africa compared to the rest of the world [2]. Furthermore, hospital case fatalities among those with heart failure range from 9% to 12.5%. This situation makes heart failure one of the major causes of death of cardiovascular origin in Africa [7]. Congestive heart failure has been recognized in Sub-Saharan Africa (SSA) for more than 60 years [8] and, based on the ageing of the population and the adoption of Western lifestyles, the African Union estimates that the current prevalence of CHF is 10–20 million people in sub-Saharan Africa, making it the leading health challenge after AIDS [9]. Since the 1950s, the significant contributions of hypertensive heart disease, cardiomyopathy, and rheumatic heart disease (RHD) as competing risks to CHF in young African adults have been recognized [10]. Moreover, atrial fibrillation, ventricular arrhythmias, stroke, thromboembolism, delirium, hypotension, myocardial infarction, acute renal failure, acute respiratory failure, cardiogenic shock, pneumonia, and pulmonary edema were among the most common complications of CHF [11–13].

Approximately 9% of all deaths in Ethiopia are attributable to CHF [14]. A systematic review of studies in Ethiopia between 1960 and 2011 showed that CHF was the cause of 4 to 24%, 8.9 to 9.8%, and 6.5 to 24% of morbidity, intensive care unit admission, and mortality, respectively [15]. Another systematic review found that 2.5% of deaths among all age groups in a sampled hospital-based mortality study were attributable to congestive heart failure [16]. Moreover, based on surveillance data analysis in Addis Ababa, congestive heart failure is the third leading cause of cardio-vascular death, following hypertension and stroke [17]. In Tikur Anbessa specialized hospital medical ward to evaluate the severity of heart disease, it was found that myocardial infarction, stroke, and high blood pressure account for about 75% of CHF deaths. Modifiable risk factors like smoking, high cholesterol, and high blood pressure explain the major share of the CHF burden in Ethiopia [18]. In this case, the results may not be directly generalizable to patients who were dying with complications from CHF, so knowing and preventing complications from CHF is vital. Identifying the factors that affect the survival of CHF patients is absolutely essential to take action for earlier treatment and to prolong the survival of CHF patients. According to several studies from around the world, there is an increase in CHF illness complications, especially in developing countries [9, 14]. However, research on the variables that affect the survival and complications of CHF patients has been limited in Ethiopia. The Felege-Hiwot referral hospital is one of the biggest tertiary-level referral hospitals in the region. The referral hospital has more than 400 beds, nine operating tables, and a diagnostic laboratory for HIV testing, as well as different clinical specimens are processed using the appropriate standard procedures. Given this context, the aim of this study was to identify the factors affecting the survival time to complications of congestive heart failure patients treated from January 2016 to December 2019 at Felege Hiwot comprehensive specialized referral hospital using the Cox proportional hazards model.

## Materials and methods

### Study setting, data source, and study design

A hospital-based retrospective follow-up study was conducted among congestive heart failure (CHF) patients under the outpatients’ clinic from January 2016 to December 2019 at Felege Hiwot comprehensive specialized referral hospital, Bahir Dar, Ethiopia. This hospital provides an organized CHF follow-up care program with mostly regional and national full-sized laboratory equipment.

### Operational definitions

#### Complication

Clinical complications of congestive heart failure are unanticipated problems that occur as a result of a procedure, treatment, or other illness, such as sudden cardiac death, renal failure, hypotension, liver damage, heart valve problems, kidney damage or failure, stroke, lung complications, and recurrent nosocomial infections as a result of frequent hospitalizations and central venous access. These complications make the function of the heart difficult and involve a worsening in the severity of the congestive heart failure diseases by causing unintentional weight loss (cardiac cachexia), dysfunction (cardio renal disease), and liver dysfunction (hepatic congestion) and lead the patients to death.

#### New York Heart Association class

The New York Heart Association (NYHA) symptomatic functional classification provides a simple way of classifying the extent of heart failure. It places patients in one of four categories based on how much they are limited during physical activity; the limitations and symptoms are in regard to normal breathing and varying degrees of shortness of breath and/or angina.

### Study participants, sampling technique and sample size

The study population was comprised of CHF patients receiving CHF treatment at the Felege Hiwot comprehensive specialized referral hospital. A simple random sampling method was adopted for selecting a representative sample from the list of medical charts that contained the list of CHF patients’ identification numbers, and patients were selected randomly using their unique identification numbers. The study excluded those patients who were complicated before the study period. The appropriate sample size was determined by the following formula [19–21] as:

$$n={\frac{(Z_{\beta \;}\;+\;Z_{\frac{\alpha }{2}})\;}{P_{1}*P_{0\;}*\gamma^{2}}}^{2}$$

Where *n* = the sample size required, *α* = the level of significance, *β* = the power of the test, *γ* = the regression coefficient representing effect of low the two groups, *P*_0_ = the probability of an event occurring in the first group and *P*_1_ = the probability of an event occurring in the second group. In this study we used 80% (*Z*_*β*_ = 0.84) power of a test and 5% ($Z_{\frac{\alpha }{2}}=1.96$) level of significance from the standard normal distribution table. The values for *P*_0_, *P*_1_, and *γ* were taken from a previous study done at the University of Gondar referral hospital by [22], and the authors categorized patients as hyponatremia (group one) if their blood sodium level was < 135 mmol/L and normonatremia (group two) if it was ≥ 135 mmol/L at their first admission to the internal medicine department. The study also found that 24.75% (*P*_0_) and 42.1% (*P*_1_) of heart failure patients died within the study period from hyponatremia and normonatremia groups, respectively. In addition, *γ*, the regression coefficient, represents the relative effect of low blood sodium level on the survival time of CHF patients as compared to normal sodium level, and its value is extracted as the log of the hazard ratio of hyponatremia to normonatremia patients (*log*4.003 = 0.602). Thus, the sample size was calculated using the above formula and a 95% confidence level. The total sample size was 218 after adding 5% for the non-response rate.

### Variables in the study

In this study, we considered two types of variables, namely, outcome and explanatory variables.

#### Outcome variable

The response variable considered in this study is the time to developing complications of heart failure patients in months like cardiac cachexia, impaired kidney function, hepatic congestion, cardio renal disease, hypotension, heart valve problems, stroke, lung complications, and recurrent nosocomial complications. The data was extracted by subtracting the date of the first visit due to CHF from the date of the first visit with complications.

#### Explanatory variables

The predictor variables that were included in this study were socio-demographic characteristics of CHF patients and the history of epidemiological, clinical, and laboratory-related factors, as summarized in Table 1.

**Table 1:** Predictor variables considered in the study.

Predictors	Category	Measurement
Gender	Female, Male	Categorical
Residence	Rural, Urban	Categorical
Age		Year
New York heart association	Class I, Class II, Class III, Class IV	Categorical
Sodium		mmol/L
Hemoglobin level		g/dL
Left ventricular ejection fraction		%
Albumin		g/dL
Thyroid stimulating hormone		mIU/L
Platelets		mcL
Creatinine		mg/dL
White blood cell		mcL
Potassium		mEq/L

Key: mmol/L (millimole per liter); g/dl (gram per deciliter); mIU/L (milli international unit per liter); mcL (micro liter); mEq/L (milli equivalent per liter); mg/dl (milli gram per deci liter).

### Data processing and editing

Checking the consistency of the data, editing, labeling, treating missing values that exist in the dataset and descriptive analysis were conducted through SPSS software Version 26. For further analysis of the data, we exported the data to the R statistical software. Variable selection was carried out using the purposeful variable selection method, where the uni-variable analysis was done at a 25% level and the multi-variable analysis at a 5% level of significance.

### Methods of statistical data analysis

Survival analysis is the analysis of statistical data in which the outcome variable of interest is the time until an event occurs. Comparing two or more estimated survival curves is the most frequently used statistical tool in recent clinical research [23]. The study used the Kaplan-Meier curves and the log-rank test for comparing the survival times obtained from two or more groups [24]. On the other hand, the Cox proportional hazard model is used to identify the relationship between the survival experience of an individual and explanatory variables [25].

#### Cox proportional hazard model and parameter estimation

The basic model for survival data is the proportional hazards (PH) model, proposed by Cox (1972) [26]. From this, estimates of the survivor function and hence the median survival time can be obtained. Although the model is based on the assumption of proportional hazards, no particular form of probability distribution is assumed for the survival times. The model is therefore referred to as a semi-parametric model. In this model, the risk of complication at time t can be expressed as: h (t,x,β) = *h*_0_ (t) r(x,β). Moreover, Cox (1972) suggested that r(x,β) = exp(xβ), with this parameterization the hazard function is h(t,x,β) = *h*_0_ (t)exp(xβ), and the hazard ratio is:

$$HR(t,\;x_{1},\;x_{0})=\frac{h(t,x_{1},\beta )}{h(t,x_{0},\beta )}=e^{\beta (x_{1}-x_{0})}$$

Where; HR: hazard rate, *h*_0_(*t*): the baseline hazard function that characterizes how the hazard function changes as a function of survival time; h(t,x,β): The hazard function at time t with covariates *x* = (*x*_1_, *x*_2_,…,*x*_*p*_) and a column vector of regression parameters *γ* = (*β*_1_, *β*_2_,…,*β*_*p*_) and *t* = the complication time, where all values of the covariates are zero, ***i*.*e*.** *r*(*x* = 0, β) = 1.

This model is used to determine which combinations of the potential explanatory variables affect the form of the risk of complications and to obtain an estimate of the hazard function itself for an individual. The term "proportional hazards" means the hazard functions are multiplicatively related (*i*.*e*., their ratio is constant over survival time). In many clinical trials, most of the time-varying covariates are biomarkers. These are always endogenous covariates. With these types of covariates, there is a usual measurement error (*i*.*e*., biological variation), the complete history is not available, and their existence is directly related to the failure status of patients. Therefore, the extended Cox model is valid only for exogenous time-dependent covariates. Treating those endogenous covariates (like biomarkers) as exogenous covariates may produce spurious results [27]. After a model is fitted, the adequacy of the fitted model needs to be assessed. The methods that involved model checking for this study used evaluation of the parametric baselines and the Cox-Snell residuals [28]. Maximum likelihood estimation (MLE) technique is used for estimating parameters that can be obtained by maximizing the joint probability (likelihood function) for the values of the data. Cox regression relies on underlying assumptions like the linearity and additivity of predictor variables, as do statistical tests and models in general. The fundamental assumption of the Cox model is that the hazards are proportional (PH), meaning that the relative risk stays constant across time with varying levels of predictor or covariate. Consequently, conducting multi-collinearity tests is not required when using Cox proportional modeling [29]. For this analysis, repeated measure data were used, and the last observation carried forward (LOCF) approach was used to replace any missing information for biomarkers [30].

## Ethical considerations

Ethical clearance was obtained and waived to collect relevant data for this study from the Research Ethics and Community Service Review Committee of the College of Science, Bahir Dar University. Due to the nature of the study (retrospective data collection), ethical approval for consulting the patient’s informed consent was not deemed necessary. All information obtained from the medical charts of study participants was coded to maintain confidentiality.

## Results

In this study, we examined the 218 CHF patients who were treated at Felege Hiwot comprehensive specialized referral hospital to recover from the disease and check for the incidence of complications continuously. Among the sampled patients included in the study, more than three-fourths of patients 194 (84.99%) had developed complications, whereas 24 (11.01%) were censored. Of the participants, 43.6% were male. Most of the 151(69.4%) congestive heart failure patients reside in urban areas. On the other hand, a large proportion of patients were classified as NYHA IV (44%). According to the descriptive result, 87.7% of female CHF patients developed complications, while 95.4% of urban residents also developed CHF complications. In addition, 95.8% of patients in NYHA class IV developed complications (Table 2).

**Table 2:** Results of the descriptive statistics for categorical variables.

Variables	Categories	Survival status		Total (%)
		Complicated (%)	Censored (%)	
Gender	Male	86(90.5)	9(9.5)	95(43.6)
	Female	108(87.8)	15(12.2)	123(56.4)
Residence	Rural	50(74.6)	17(25.4)	67(30.7)
	Urban	144(95.4)	7(4.6)	151(69.3)
NYHA	Class I	24(63.2)	14(36.8)	38(17.4)
	Class II	34(94.4)	2(5.6)	36(16.5)
	Class III	44(91.7)	4(8.3)	48(22)
	Class IV	92(95.8)	4(4.2)	96(44)

The descriptive results for continuous variables are given in Table 3. The average, minimum, and maximum age (in years) of CHF patients treated at Felege Hiwot referral hospital was 50.58, 16, and 82, respectively. The result also shows the average serum hemoglobin concentration (g/dl), serum sodium concentration (mmol/L), and left ventricular ejection fraction (%) were 13.19, 122.03, and 48.47, respectively. Moreover, the median time to complications for CHF patients was 22 months and the standard deviation was 14.04, with a minimum and maximum complication time of three (3) and forty-eight (48) months, respectively (Table 3).

**Table 3:** Results of the descriptive statistics for continuous variables.

Variable	Mean	Standard deviation	Minimum	Maximum
Age (Year)	50.58	20.27	16.0	82.0
Creatinine (mg/dl)	0.95	1.40	0.1	20.0
Sodium (mmol/L)	122.03	44.554	40.32	194.45
Hemoglobin (g/dl)	13.19	5.42	4.73	21.5
Albumin (g/dL)	2.84	4.19	0.25	6.19
Platelets (mcL)	167469.50	122896.36	99295.41	729000
White blood cell (mcL)	14994.64	2526.01	3482.86	22220.07
TSH (mIU/L)	6.73	76.85	0.0029	12.48
LVEF (%)	48.47	24.97	11	88.0
Potassium (mEq/L)	5.15	5.76	1.002	6.07
Time to complication (Months)	Mean = 23.4	14.04	3	48.0
	Median = 22			

### Kaplan Meier estimate of survival function for categorical variables

**Fig 1:**
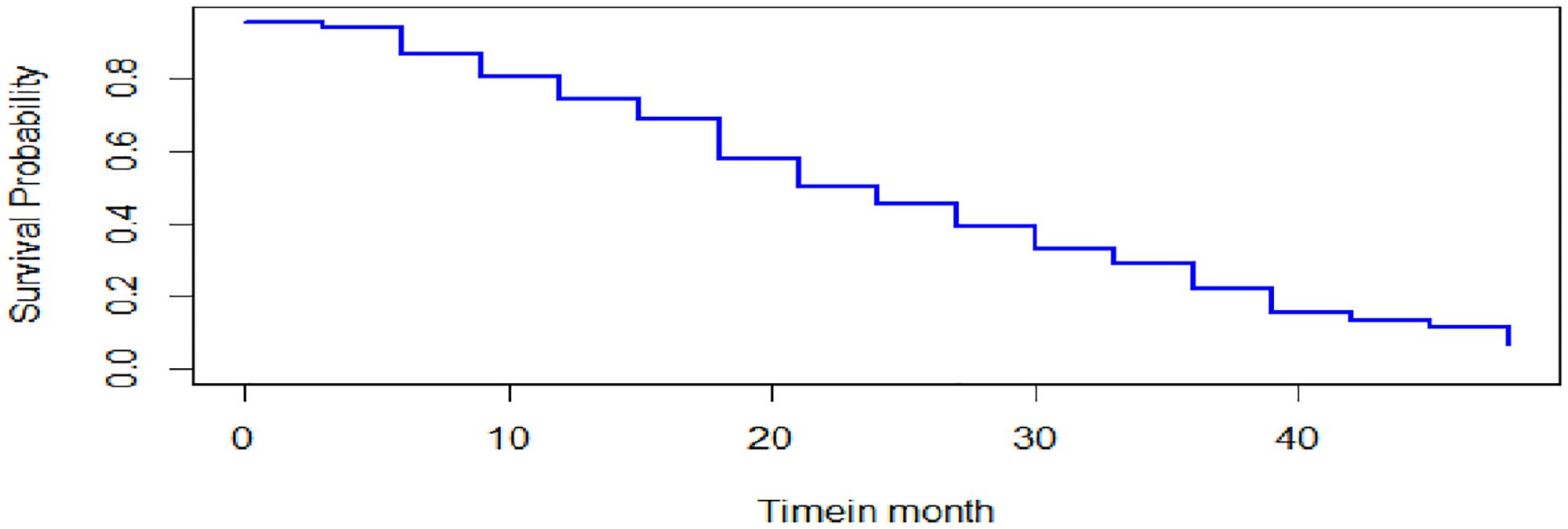
Overall Kaplan Meier estimate of survival function.

**Fig 2:**
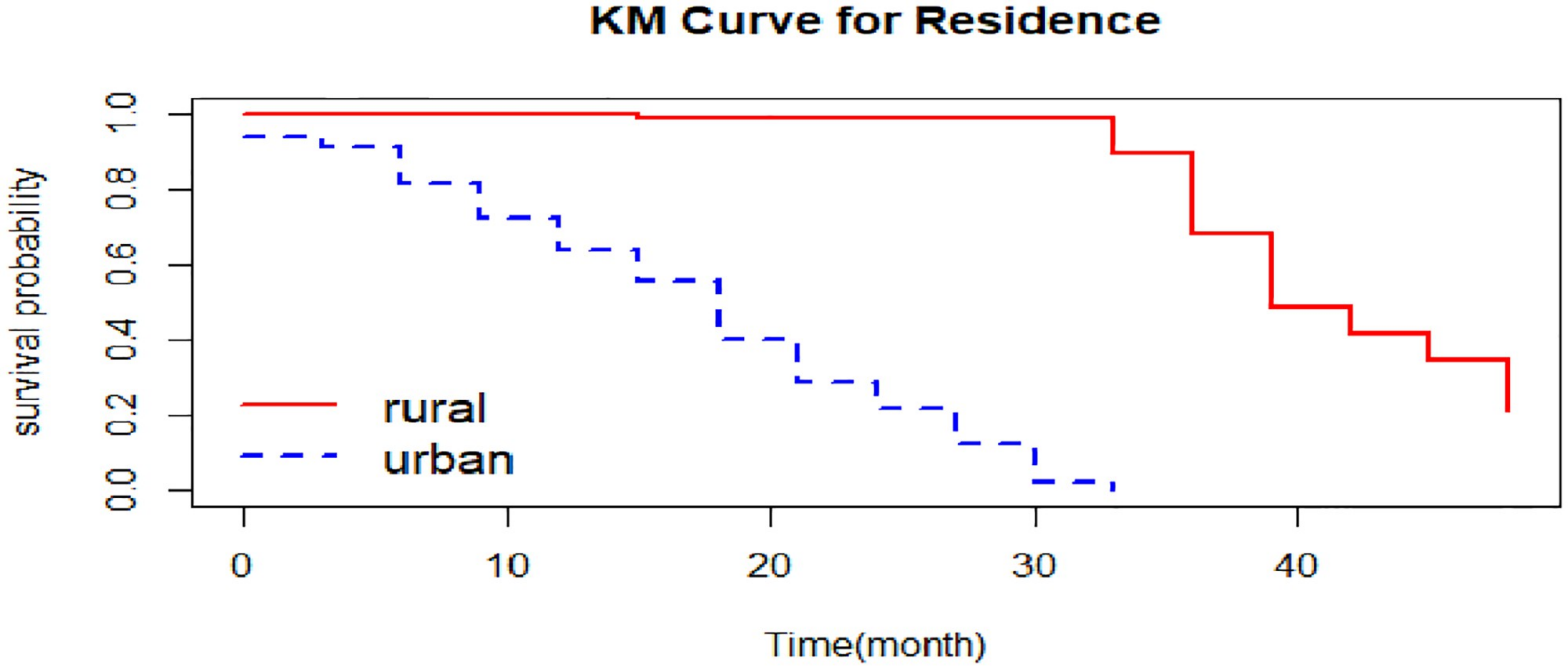
The K-M survival function for complication time of CHF patients by residence.

**Fig 3:**
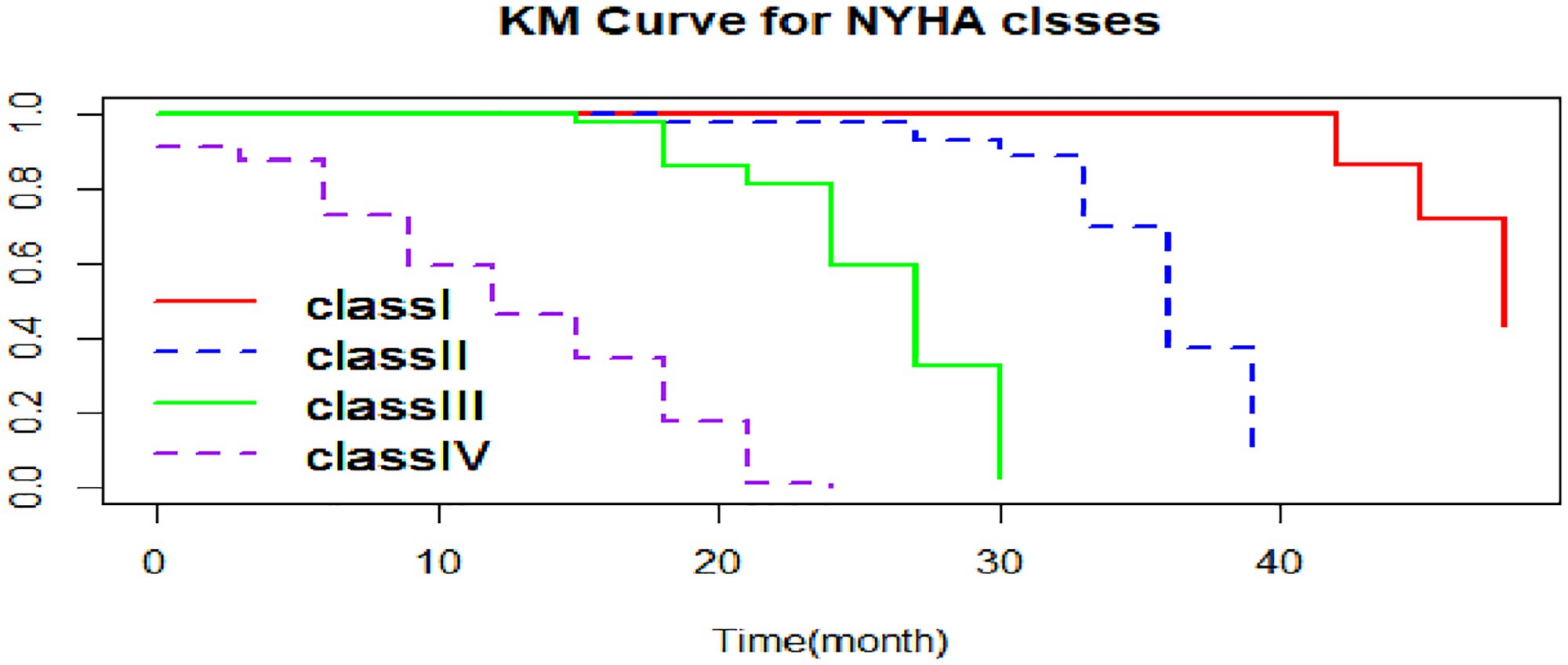
The K-M survival function for complication time of CHF patients by NYHA classes.

The overall Kaplan-Meier graph indicated in Fig 1 indicates that the estimate of survivor function indicates that CHF patients developed complications after three months from their first visit, and their survival probability decreased over the survival time. As shown in Fig 2, CHF patients in rural areas had less time to develop complications than those in urban areas. Moreover, Fig 3 indicates that patients from NYHA classes I, II, and III have recently developed the complication as compared to their counterparts in class IV.

### Results of the log-rank test

The log rank test showed that, there is a survivor time difference among the groups of patients from rural and urban areas, as well as the patients in NYHA classes I, II, III, and IV. There is a statistically significant difference between patients place residence in terms of developing time to complication that the likelihood of developing complications was higher for those CHF patients who resided in urban areas compared to those in rural areas (p-value < 0.0001). Furthermore, there is a statistically significant difference between NYHA classes (p-value < 0.0001) and showed that as the NYHA class (I to IV) increases, the chances of developing complication for CHF patients will be increased. But the test revealed that there was no survivor (complication) time difference among genders (Table 4).

**Table 4:** Results of the log-rank test for categorical variables.

Variables	Test Statistic	Standard error	Chi-Square	P-value
Residence	-68.078	5.74	140.7	<0.0001
NYHA class	-179.4	12.72	199.0	<0.0001
Gender	0.89	6.90	0.02	0.8976

### Model selection and adequacy

The global test was performed to test the hypothesis that the hazard ratios are constant over time as opposed to not constant over time. The global test was statistically insignificant; p-value = 0.433 greater than the tabulated level of significance (0.05). The assumption of constant hazard ratio over time was satisfied due to this; the Cox PH model was adequate for this data (Table 5).

**Table 5:** Proportional hazards assumption.

Variables	Chi square	P-value
sex	0.12	0.726
Age	0.23	0.635
Creatinine	1.39	0.239
Residence	0.05	0.824
Sodium	0.02	0.888
Hemoglobin	0.01	0.928
Albumin	0.55	0.459
NYHA	0.82	0.366
LVEF	0.31	0.575
WBC	0.06	0.807
TSH	1.19	0.276
Platelets	2.52	0.112
Potassium	5.11	0.024
Global	13.20	0.433

#### Results of the Cox proportional hazards model

A multivariable analysis of the Cox PH model was done by using all significant predictors in the univariable analysis. The Cox PH model revealed that age of respondents, place of residence, NYHA class, LVEF, serum sodium concentration, and serum hemoglobin concentration were found to be significantly associated factors for time to complications in CHF patients. On the other hand, serum platelet count and serum creatinine concentration were not statistically significant predictors of time to complication in CHF patients. The results are summarized in Table 6. The total follow-up time of patients recorded in the specified study time is 48 months. The current study showed that the median complication time for CHF patients was 22 months [95% CI: 21–28]. This indicates that the estimated probability that a CHF patient will survive without a complication for 22 months or more is 0.5; *i*.*e*., the smallest event time such that the probability of getting a complication earlier is greater than 0.5 is 22 months. Moreover, 12 months and 36 months were the smallest times such that the probability of getting a complication earlier was greater than 0.25 and 0.75 respectively.

**Table 6:** Result from the fitted Cox PH model.

Factors		Estimate (Hazard ratio)	Std.Error	Chi-square	P-value	95% CI for Hazard ratio	
Age		0.2464(1.28)	0.0666	13.687	0.0002	1.12	1.46
Left ventricular ejection fraction		-0.2953(0.74)	0.0655	20.3256	<0.0001	0.65	0.85
New York health association classes (reference = class IV)							
	class I	-0.8285(0.44)	0.0948	76.4332	<0.0001	0.36	0.53
	class II	-0.6213(0.54)	0.0713	75.8491	<0.0001	0.47	0.62
	class III	-0.3207(0.73)	0.0578	30.7649	<0.0001	0.65	0.81
Sodium		-0.0567(0.94)	0.0271	4.3775	0.0362	0.90	1.00
Hemoglobin		-0.1115(0.89)	0.0241	21.3543	<0.0001	0.85	0.94
Creatinine		-0.0413(0.96)	0.0595	0.4816	0.4877	0.85	1.08
Platelets		-0.4950(0.61)	0.7056	0.4922	0.4829	0.15	2.43
Residence (reference = urban)							
Rural		-0.2858(0.75)	0.0539	28.1424	<0.0001	0.68	0.84
95% Confidence interval for median time to complication (in months)						21	28

As the age of the CHF patients increased by one year, the estimated hazard ratio, HR = 1.28 [95% CI: 1.12–1.46, p = 0.0002], showed that the risk of developing complications in CHF patients increased by 28% as their age increased by a year. A 1% increase in left ventricular ejection fraction had an estimated hazard ratio of HR = 0.74 [95% CI: 0.65–0.85, p < 0.0001], implying that complications among CHF patients were reduced by 26%. A g/dl increase in serum hemoglobin concentration had an estimated hazard ratio of HR = 0.89 [95% CI: 0.85–0.94, p < 0.0001], implying that complications among CHF patients were reduced by 11%.

The estimated hazard rate of CHF patients with NYHA classes I, II, and III symptoms had HR = 0.44 [95% CI: 0.36–0.53, p < 0.0001], HR = 0.54 [95% CI: 0.47–0.62, p < 0.0001] and HR = 0.73 [95% CI: 0.65–0.81, p < 0.0001], respectively. This means that the chance of developing a complication for those patients in NYHA classes I, II, and III was 0.44, 0.54, and 0.73 times less risk of complication than for CHF patients with NYHA class IV symptoms, respectively. In addition, the estimated hazard ratio for serum sodium concentration was HR = 0.94 [95% CI: 0.90–1.00, p = 0.0362]. This indicates that at one mmol/L increment in serum sodium concentration, the risk of complications among CHF patients was significantly decreased by 6%. Moreover, the hazard of patients from rural areas was HR = 0.75 [95% CI: 0.68–0.84, p < 0.0001], which implies that the risk of complications among rural CHF patients was 0.75 times less than the risk of complications among patients from urban areas (Table 6).

## Discussion

In this study, 218 CHF patients at Felege Hiwot comprehensive specialized referral hospital, Bahir Dar, Ethiopia were assessed, of which 194 (88.99%) developed complications. The median duration of complication time of CHF patients was 22 months [95% CI: 21–28]; this showed that close monitoring of patients is vital to prolonging their survival by reducing other complications.

This study identified that the age of patients was identified as a significant predictor of the complication rate of CHF. This could be explained by the fact that as the patients aged, the risk of developing complications in CHF patients increased. In other words, the risk of complications for elderly patients was high. This result was consistent with studies [31–37].

The current study revealed that the left ventricular ejection fraction is associated with the time to complication in CHF diseases [32, 38]. This might indicate that the left ventricular ejection fraction is important for describing and diagnosing if the left side of the heart loses its ability to contract, making it unable to supply the body’s oxygen and nutrient requirements, and for framing recommendations on heart function.

In addition, the results of this study showed that the change in serum hemoglobin concentration has an impact on the development of complications in CHF diseases. The possible justification for this result could be that patients with CHF who have hemoglobin concentrations above or below the normal range, especially in the middle and older age groups, are at risk of developing other complications as a result of the decrease or increase in hemoglobin concentration. This will increase the risk of developing cardiovascular diseases. This result is also consistent with these studies [32, 35–37].

The findings of this study indicate that serum sodium concentration is a determinant of the complication rate of CHF disease. This indicates that the survival of CHF patients with a serum sodium concentration of 135 mmol/L (normonatremia) was higher than that of patients with a serum sodium concentration less than the threshold. The loss of sodium may lower plasma osmolality and the ability to completely prevent interstitial fluid from re-entering the bloodstream, resulting in patients developing complications sooner. This result was consistent with a study done by using CHF patients admitted to the Institute of Cardiology and allied hospitals in Faisalabad, Pakistan, and a CHF case study and registry in the Tobacco District [32, 37, 39].

The findings of this study showed that the place of residence affects the survival complication time of CHF patients. The possible explanation might be that those CHF patients from rural areas are not mostly exposed to cigarette smoke, recreational drugs, or heavy alcohol use and their daily activities are naturally richer in physical activity, which lowers the risk of acquiring cardiovascular disorders. This result is in line with the previous findings made by using heart failure in rural and urban patients [40].

Furthermore, the results of this study showed that the NYHA class of CHF patients is identified as a significant factor of survival time to complication. This result was consistent with previous studies [33, 35, 39, 41]. However, most of the studies done on the survival of CHF patients showed that serum creatinine concentration and serum platelet count were statistically significant [22, 32, 35–37, 42], but it contradicts to these studies; this contradiction may be due to the fact that the primary end point of those studies was mortality, but the underlying study end point was complication. In addition, the sample size considered may also be a factor for contradiction.

In conclusion, the median complication time of congestive heart failure patients was 22 months and close monitoring of patients is vital to prolonging their survival by reducing other complications. The results of this study using the Cox PH model revealed that age of respondents, place of residence, NYHA class, LVEF, serum sodium concentration, and serum hemoglobin concentration were found to be significantly associated factors for time to complications in CHF patients. The overall KM curve for the survival of CHF patients shows that the survival probability of CHF patients is a decreasing step function with a corresponding increment in their survival times. Thus, we recommend that healthcare awareness be given about the causes of the complications earlier for those aged patients and CHF patients with serum sodium concentrations of less than the threshold value to reduce the risk of developing complications which might result in mortality in congestive heart failure patients.

## Limitations

In this study, in the Cox PH model, the authors did not see the interaction effect of the predictors over time, so anyone may assess the clinical importance of their interaction throughly.

## Supporting information

S1 Data (SAV)

## Abbreviations

AIDS: Acquired Immune Deficiency Syndrome
CHF: Congestive Heart Failure
CI: Confidence Interval
CVD: Cardiovascular Diseases
HIV: Human Immune Virus
HR: Hazard Ratio
KM: Kaplan Meier
LVEF: Left Ventricular Ejection Fraction
MLE: Maximum Likelihood Estimation
NYHA: New York Heart Association
PH: Proportional Hazard
RHD: Rheumatic Heart Diseases
SPSS: Statistical Package for Social Science
SSA: Sub-Saharan Africa
Std.Error: Standard Error
TSH: Thyroid Stimulating Hormone
WBC: White Blood Cell
WHO: World Health Organization
